# Psoriatic arthritis and psoriasis have comparable cardiovascular outcomes: Results from a TriNetX Global Network cohort analysis

**DOI:** 10.1016/j.jdin.2025.11.015

**Published:** 2025-11-29

**Authors:** Laurent Fauchier, Thibault Lenormand, Lisa Lochon, Mickael Guglieri

**Affiliations:** Service de Cardiologie, Centre Hospitalier Universitaire Trousseau et Faculté de Médecine, Université François Rabelais, Tours, France

**Keywords:** cardiovascular risk, heart failure, inflammation, myocardial infarction, psoriasis, psoriatic arthritis

*To the Editors:* Psoriasis and psoriatic arthritis (PsA) are chronic immune-mediated diseases associated with systemic inflammation and increased cardiovascular morbidity.^1^^,^^2^ Although both share pathogenic pathways involving interleukin-17, interleukin-23, and tumor necrosis factor alpha, it remains unclear whether PsA confers additional cardiovascular risk beyond that of psoriasis alone.^3^^,^^4^ Prior cohort studies suggest elevated rates of major adverse cardiovascular events in both groups compared with the general population, but results directly comparing PsA and psoriasis have been variable after adjustment for traditional risk factors. Using the large, real-world TriNetX network, we aimed to clarify differences in cardiovascular outcomes between patients with PsA and psoriasis-only.

Using de-identified electronic health records from the TriNetX Global Collaborative Network (156 health care organizations; >130 million patients), we identified adults (≥18 years) diagnosed with PsA between 2010 and 2024 (International Classification of Diseases, Tenth Revision, Clinical Modification [ICD-10-CM L40.5]) and compared them to patients with psoriasis without PsA (ICD-10-CM L40).^5^ Propensity-score matching (1:1) was performed across 67 demographic, clinical, laboratory, and treatment variables, including disease-modifying antirheumatic drugs (DMARDs) (Table I). Outcomes included all-cause death, ischemic stroke or thromboembolism, acute myocardial infarction, incident atrial fibrillation, ventricular arrhythmias/cardiac arrest, heart failure, pericarditis, and myocarditis. Events were assessed from 45 days to 5 years after the index diagnosis. Hazard ratios and 95% confidence intervals were calculated within TriNetX using Kaplan–Meier survival analyses and Cox proportional hazard models. Given the number of outcomes assessed, *P* values were adjusted for multiple comparisons using the Benjamini–Hochberg false discovery rate method. Analyses complied with Health Insurance Portability and Accountability Act and TriNetX de-identification standards.

**Table I:** Baseline characteristics of patients with psoriasis based on the presence or not of psoriatic arthritis

Variables for characteristics	Before propensity-score matching			After propensity-score matching		
	Psoriatic arthritis	No psoriatic arthritis	Std diff. (%)	Psoriatic arthritis	No psoriatic arthritis	Std diff. (%)
	(n = 119,022)	(n = 317,592)		(n = 102,385)	(n = 102,385)	
Demographics						
Age (y), mean ± SD	53.0 ± 14.8	50.8 ± 18.2	13.1	52.9 ± 14.9	53.3 ± 16.8	2.8
Men, n (%)	48,485 (40.7%)	146,798 (46.2%)	11.1	42,800 (41.8%)	43,234 (42.2%)	0.9
White, n (%)	93,827 (78.8%)	229,884 (72.4%)	15.1	80,337 (78.5%)	80,356 (78.5%)	0
Black or African American, n (%)	4281 (3.6%)	18,827 (5.9%)	11	3860 (3.8%)	3917 (3.8%)	0.3
Risk factors and cardiovascular comorbidities						
Hypertension, n (%)	38,995 (32.8%)	102,798 (32.4%)	0.8	33,848 (33.1%)	33,553 (32.8%)	0.6
Diabetes mellitus, n (%)	17,669 (14.8%)	44,979 (14.2%)	1.9	15,364 (15%)	15,081 (14.7%)	0.8
Smoker, n (%)	10,367 (8.7%)	25,329 (8%)	2.7	8891 (8.7%)	8649 (8.4%)	0.8
Overweight or obesity, n (%)	20,757 (17.4%)	51,865 (16.3%)	3	17,706 (17.3%)	17,297 (16.9%)	1.1
Dyslipidemia, n (%)	33,986 (28.6%)	96,395 (30.4%)	3.9	29,759 (29.1%)	29,090 (28.4%)	1.4
Heart failure, n (%)	4047 (3.4%)	11,782 (3.7%)	1.7	3553 (3.5%)	3455 (3.4%)	0.5
Coronary artery disease, n (%)	8701 (7.3%)	23,697 (7.5%)	0.6	7610 (7.4%)	7461 (7.3%)	0.6
Ischemic stroke, n (%)	1546 (1.3%)	4785 (1.5%)	1.8	1362 (1.3%)	1349 (1.3%)	0.1
Atrial fibrillation or flutter, n (%)	4380 (3.7%)	12,532 (3.9%)	1.4	3881 (3.8%)	3837 (3.7%)	0.2
Noncardiovascular comorbidities						
Kidney disease, n (%)	7542 (6.3%)	21,006 (6.6%)	1.1	6626 (6.5%)	6646 (6.5%)	0.1
COPD, n (%)	4480 (3.8%)	14,459 (4.6%)	4	3975 (3.9%)	3881 (3.8%)	0.5
Sleep apnea syndrome, n (%)	12,315 (10.3%)	26,306 (8.3%)	7.1	10,240 (10%)	10,047 (9.8%)	0.6
Laboratory tests and examinations						
Body mass index (kg/m^2^), mean ± SD	31.6 ± 7.8	30.3 ± 7.7	17.1	31.4 ± 7.7	31.2 ± 7.9	2.2
Estimated GFR (MDRD, mL/min), mean ± SD	82.6 ± 24.5	82.7 ± 26.4	0.3	82.6 ± 24.9	82.7 ± 25.2	0.5
Medications						
β-Blockers, n (%)	20,064 (16.9%)	49,557 (15.6%)	3.4	16,563 (16.2%)	16,204 (15.8%)	1
Calcium channel blockers, n (%)	12,367 (10.4%)	32,629 (10.3%)	0.4	10,265 (10%)	10,068 (9.8%)	0.6
ACE inhibitors, n (%)	12,688 (10.7%)	35,242 (11.1%)	1.4	10,594 (10.3%)	10,437 (10.2%)	0.5
Angiotensin II inhibitors, n (%)	10,141 (8.5%)	24,487 (7.7%)	3	8322 (8.1%)	8165 (8%)	0.6
Diuretics, n (%)	18,135 (15.2%)	47,290 (14.9%)	1	14,922 (14.6%)	14,583 (14.2%)	0.9
Lipid-lowering drugs, n (%)	22,877 (19.2%)	61,059 (19.2%)	0	19,004 (18.6%)	18,580 (18.1%)	1.1
Glucose-lowering therapy, n (%)	14,840 (12.5%)	37,112 (11.7%)	2.4	12,329 (12%)	12,070 (11.8%)	0.8
Antiplatelet therapy, n (%)	13,799 (11.6%)	36,965 (11.6%)	0.1	11,424 (11.2%)	11,159 (10.9%)	0.8
Methotrexate, n (%)	21,305 (17.9%)	9118 (2.9%)	50.8	8683 (8.5%)	8983 (8.8%)	1
Sulfasalazine, n (%)	5614 (4.7%)	1251 (0.4%)	27.7	1591 (1.6%)	1247 (1.2%)	2.9
Hydroxychloroquine, n (%)	5244 (4.4%)	3309 (1%)	20.8	2924 (2.9%)	2518 (2.5%)	2.5
Leflunomide, n (%)	3302 (2.8%)	726 (0.2%)	21	1057 (1%)	725 (0.7%)	3.5

Values are n (%) or mean ± SD. *ACE*, Angiotensin-converting enzyme; *COPD*, chronic obstructive pulmonary disease; *GFR*, glomerular filtration rate; *MDRD*, modification of diet in renal disease; *Std diff.*; standardized difference.

Among 446,614 adults with psoriasis identified in TriNetX (2010-2024), 119,022 had PsA. After 1:1 propensity-score matching, both cohorts (n = 102,385 per group) were well balanced on baseline characteristics (Table I). The mean age was 53 years, 42% were men, and major comorbidities included hypertension (33%), diabetes (15%), dyslipidemia (29%), and obesity (17%). During a median follow-up of 3.7 years, after matching only for age, sex, and race, PsA was associated with higher risks of ischemic stroke, atrial fibrillation, heart failure, and pericarditis compared with psoriasis alone (Table II). After matching for all cardiovascular risk factors, comorbidities, and concomitant drugs (excluding DMARDs), patients with PsA showed a globally lower risk of cardiovascular outcomes, including all-cause death, myocardial infarction, and arrhythmias. When DMARDs were included in the matching model, risks became overall similar between groups.

**Table II:** Clinical outcomes during follow-up in the matched populations (follow-up , 3.7 ± 1.6 years; median, 4.8; IQR, 2.7)

Clinical outcomes	Psoriatic arthritis		No psoriatic arthritis		Hazard ratio (95% CI)	*P* value	Adjusted *P* value
	No. of events	Yearly rate, %	No. of events	Yearly rate, %			
Matching on age, sex, race, and socioeconomic status	n = 117,384		n = 117,384				
Death	3788	0.92	4627	1.08	0.836 (0.801-0.873)	<.0001	<.0001
Ischemic stroke or thromboembolism	2315	0.57	2203	0.53	1.071 (1.010-1.135)	.02	.04
Acute MI	869	0.20	942	0.21	0.936 (0.854-1.027)	.16	.24
Incident AF	3906	0.97	3681	0.90	1.083 (1.036-1.133)	<.0001	<.0001
VT/VF/cardiac arrest	1786	0.42	1882	0.43	0.964 (0.904-1.029)	.27	.35
Incident HF	4730	1.17	4435	1.08	1.087 (1.044-1.133)	<.0001	<.0001
Acute pulmonary edema or cardiogenic shock	833	0.20	870	0.20	0.977 (0.888-1.074)	.63	.63
Pericarditis	1085	0.26	963	0.23	1.149 (1.053-1.253)	.002	.005
Myocarditis	86	0.02	78	0.02	1.123 (0.827-1.526)	.46	.52
Matching on all variables of Table 1 (excluding DMARD)	n = 120,407		n = 120,407				
Death	4359	1.01	5720	1.30	0.768 (0.738-0.799)	<.0001	<.0001
Ischemic stroke or thromboembolism	2365	0.56	2470	0.58	0.962 (0.910-1.018)	.18	.23
Acute MI	864	0.19	988	0.22	0.877 (0.801-0.961)	.01	.02
Incident AF	4001	0.96	4419	1.06	0.907 (0.869-0.947)	<.0001	<.0001
VT/VF/cardiac arrest	1830	0.41	2100	0.47	0.875 (0.822-0.931)	<.0001	<.0001
Incident HF	4823	1.15	5251	1.25	0.92 (0.885-0.957)	<.0001	<.0001
Acute pulmonary edema or cardiogenic shock	846	0.20	928	0.22	0.918 (0.836-1.008)	.07	.11
Pericarditis	1109	0.26	1060	0.24	1.053 (0.968-1.146)	.23	.26
Myocarditis	86	0.02	93	0.02	0.928 (0.692-1.245)	.62	.62
Matching on all variables of Table 1 (including DMARD)	n = 102,385		n = 102,385				
Death	3856	1.04	4815	1.30	0.802 (0.769-0.837)	<.0001	<.0001
Ischemic stroke or thromboembolism	2070	0.58	2146	0.60	0.964 (0.907-1.024)	.23	.3
Acute MI	783	0.21	819	0.22	0.953 (0.864-1.051)	.34	.38
Incident AF	3508	0.99	3677	1.05	0.953 (0.910-0.998)	.04	.12
VT/VF/cardiac arrest	1610	0.43	1724	0.46	0.932 (0.871-0.998)	.04	.12
Incident HF	4200	1.18	4457	1.26	0.94 (0.901-0.981)	.004	.02
Acute pulmonary edema or cardiogenic shock	732	0.20	787	0.21	0.93 (0.841-1.028)	.16	.24
Pericarditis	963	0.26	883	0.24	1.091 (0.995-1.195)	.06	.11
Myocarditis	71	0.02	78	0.02	0.909 (0.659-1.254)	.56	.56

*AF*, Atrial fibrillation; *DMARD*, disease-modifying antirheumatic drugs; *HF*, heart failure; *IQR*, interquartile range; *MI*, myocardial infarction; *VF*, ventricular fibrillation; *VT*, ventricular tachycardia.

In this large real-world TriNetX analysis, the apparent cardiovascular excess in PsA compared with psoriasis alone disappeared after comprehensive adjustment for comorbidities and treatment exposures. Although limited by its retrospective electronic health record design, this large TriNetX analysis provides a more comprehensive, treatment-adjusted evaluation than prior studies. With detailed comorbidity and medication matching, it offers the most robust real-world comparison to date, showing no excess—and possibly lower—cardiovascular risk in PsA versus psoriasis.^2^, ^3^, ^4^ The lower mortality and comparable event rates observed in PsA may partly reflect earlier diagnosis, closer multidisciplinary follow-up, and more frequent and aggressive use of systemic anti-inflammatory therapies—especially DMARDs—that are aimed at joint protection but also effective in reducing systemic inflammation. Together, these data suggest that optimal and timely control of inflammation in PsA may offset its cardiovascular burden and improve long-term outcomes.

## Conflicts of interest

Dr Fauchier is consultant or speaker fees of small amounts for AstraZeneca, Bayer, BMS/Pfizer, Boehringer Ingelheim, Boston Scientific, Medtronic, Novo Nordisk, and Zoll outside of this work. Drs Lenormand, Lochon, and Guglieri have no conflicts of interest to declare.
